# Boswellic acid synergizes with low-dose ionizing radiation to mitigate thioacetamide-induced hepatic encephalopathy in rats

**DOI:** 10.1186/s40360-024-00831-w

**Published:** 2025-01-13

**Authors:** Dina E. Saad, Somaya Z. Mansour, Eman I. Kandil, Asmaa Hassan, Fatma S. M. Moawed, Mustafa M. M. Elbakry

**Affiliations:** 1 Radiation Biology Research, National Center for Radiation Research and Technology, Egyptian Atomic Energy Authority, Cairo, Egypt; 2 Biochemistry Department, Faculty of Science, Ain-Shams University, Cairo, Egypt; 3 Health Radiation Research, National Center for Radiation Research and Technology, Egyptian Atomic Energy Authority, Cairo, Egypt

**Keywords:** Hepatic encephalopathy, Boswellic acid, LDR, IL18, Paraoxonase-1, Neurotransmitters

## Abstract

Hepatic encephalopathy (HE) is a syndrome that arises from acute or chronic liver failure. This study was devised to assess the impact of a combination of boswellic acid (BA) and low doses of gamma radiation (LDR) on thioacetamide (TAA)-induced HE in an animal model. The effect of daily BA treatment (175 mg/kg body weight, for four weeks) and/or fractionated low-dose γ-radiation (LDR; 0.25 Gy, twice the total dose of 0.5 Gy) was evaluated against TAA (200 mg/kg, intraperitoneal) twice-weekly for four weeks to induce liver damage and HE in rats. TAA-exposed rats exhibited a significant elevation in serum activities of liver enzymes (GGT, ALP) and plasma ammonia levels at *P* < 0.05 (Duncan’s test) compared to the control group. Moreover, there was an increase in the levels of proinflammatory cytokines (IL6, IL12, IL18) in the TAA-exposed animals accompanied by a depletion in the activities of paraoxonase-1 and neurotransmitter contents compared with normal control rats (*P* < 0.05). However, the administration of BA alone or in combination with LDR led to improvements in liver and brain parameter indices. Furthermore, the histopathological assessments of liver and brain tissues supported the findings of the biochemical investigations. From the statistical analysis, it can be concluded that the combined administration of BA and exposure to LDR may possess potential hepatoprotective effects against hepatic encephalopathy-associated hyperammonemia and the consequent damage to the liver and brain. This study proposes that a combination of therapeutic approaches, LDR and BA could be a new therapeutic candidate for the management of hepatic encephalopathy.

## Introduction

Hepatic encephalopathy (HE) is a prevalent and severe complication associated with liver failure, often resulting in unfavorable outcomes for affected individuals. Currently, there is no definitive method for diagnosis, and the underlying mechanisms contributing to HE remain inadequately clarified [1]. In severe cases, it can lead to death and various neuropsychiatric disorders, ranging from mild cognitive deficits to intense confusion, disorientation, and even coma [2]. Increased ammonia levels in the blood play a crucial role in the onset of HE. Ammonia is primarily produced in the intestines through processes involving bacterial urease activity, the deamination of amino acids, and protein metabolism. Additionally, ammonia is generated and utilized in multiple metabolic processes occurring in the muscles, kidneys, and brain, among other organs. In cases of liver failure, the key pathway for ammonia metabolism is disrupted, resulting in hyperammonemia [3]. While the exact etiology of hepatic encephalopathy is still under investigation, many experts suggest that ammonia is a significant factor contributing to the condition [4].

The complexities in the etiology of serious diseases involve multiple targets. Consequently, conventional single-target drug therapies may yield limited benefits for patients. To achieve greater therapeutic effectiveness, it is essential to shift our focus toward developing treatments that target multiple pathways. One promising approach is the use of combination therapies or innovative combinatorial drugs, which can provide more comprehensive treatment options for complex health issues. The goal of utilizing multi-component combinations is to enhance therapeutic outcomes while minimizing potential side effects [5]. Given that these combinations interact on various levels, their overall effects can be synergistic, additive, or, in some cases, antagonistic. To ensure patient safety and maximize benefits, it is vital that multi-component combinations are rigorously tested and scientifically evaluated, as this will help mitigate the risks associated with drug interactions and provide patients with safer, more effective treatment options. Numerous studies have sought to explore safe alternative treatments, especially those derived from natural sources [6]. The growing interest in medicinal plants is driven by their lower side effects and the array of beneficial compounds they contain, as endorsed by the World Health Organization (WHO) [7]. Many researchers advocate for integrating natural medicinal compounds with low doses of radiation to minimize harmful effects while enhancing treatment efficacy for various diseases [8].

Boswellic acids (BA), which are pentacyclic triterpenic acids extracted from the resin of Boswellia species, exhibit a range of pharmacological properties, including anti-inflammatory, antioxidant, hepatoprotective, gastroprotective, antimicrobial, and anti-excitotoxic effects [9]. Reports indicate that BA could have potential therapeutic benefits for neurological disorders, owing to their capacity to reduce neurotoxic aggregates, mitigate oxidative stress, and improve cognitive function. Recent studies have also proposed BA as a promising candidate for treating brain tumors, as it may inhibit cell proliferation, angiogenesis, and metastasis, while promoting apoptosis in both laboratory and animal models [10, 11]. However, one of the limitations of using BA is poor pharmacological performance. Although radiation exposure carries some risks, these risks are reduced at lower levels. Numerous studies in biology, medicine, and epidemiology have increasingly pointed out the potential health benefits of low-dose ionizing radiation (LDR). Emerging evidence indicates that exposure to LDR may help reduce cancer rates, lower the mortality associated with cancer, enhance neurological function, and mitigate complications related to diabetes [12]. Therefore, the current study proposed that BA combined with whole-body low-dose gamma radiation (LDR), could enhance the ameliorative effects of BA against TAA-induced HE in rats.

## Materials and methods

### Chemicals

Boswellic acid (BA) was acquired from Best Naturals, USA (HY-N2513), and thioacetamide was acquired from Qualikems, India (T1035). High purity grade (98.5 %) suppurative substances were all used in this investigation.

### Animals

The National Centre for Radiation Research and Technology (NCRRT) Animal House provided 80 male Wistar Albino rats weighing between 120 and 150 g (Nasr City, Egypt). Before the commencement of the experiment, they were housed in NCRRT for around two weeks to allow for the acclimatization of laboratory conditions and rule out any concurrent infections. Throughout the trial, the animals were housed in conventional, clean laboratory settings with an ample supply of standard pellet feeds and unrestricted access to clean, fresh water to drink. The worldwide criteria for animal experimentation were followed in the care and use of laboratory animals, as approved by the Institutional Animal Ethical Committee. Additionally, the present study was approved by the Institutional Animal Care and Use Committee Research Ethic Board of the National Center for Radiation Research and Technology (REC-NCRRT) (48A/22).

### Radiation facility

Rats were given whole-body gamma irradiation at the National Centre for Radiation Research and Technology (NCRRT) in Cairo, Egypt, using Canadian Gamma Cell-40 and Cesium-137 Biological Sources. During the final two weeks of the experiment, rats were subjected to a low dosage of gamma irradiation (0.25 Gy/week for a total of 0.5 Gy/animal) [13].

### Experimental protocol

Rats were distributed equally into eight main groups (10 per group) as follows: **Group 1 (Control; C**): Rats were fed a normal diet for 4 consecutive weeks serving as normal control. **Group 2 (BA):** Rats were received orally with Boswellic acid (175 mg/kg body weight daily) for 4 weeks according to Jyothi et al. (2006). **Group 3 (LDR):** Rats were exposed to LDR (0.25 Gy/week delivered to 0.5 Gy) [13]. **Group 4 (BA+LDR):** Rats received Boswellic acid orally as mentioned in the group (2) then were exposed to LDR as in group (3). **Group 5 (TAA):** Rats were injected with thioacetamide intraperitoneally (200 mg/kg body weight) twice a week for one month [14]. **Group 6 (TAA+BA):** Rats were injected intraperitoneally with TAA as in the group (5) and then were administrated with BA as in group (2) after the last dose of TAA. **Group 7 (TAA+LDR):** Rats were injected intraperitoneally with thioacetamide as mentioned in group (5), then were exposed to LDR as in group (3) after the last dose of TAA. **Group 8 (TAA +BA+LDR):** Rats were injected with TAA as in group (5), then BA as in group (2) then LDR as in group (3) after the last dose of TAA.

### Samples collection

Rats were slaughtered under urethane anesthesia at the end of the experiment, and their hearts were punctured to remove their complete blood. To analyze ammonia, the blood was drawn on EDTA. The remaining blood samples were drawn, allowed to clot for 15 min at 37 °C, and then centrifuged for 10 min at 3000 rpm to extract serum. In preparation for additional biochemical studies, the separated serum was kept at −20 °C. Furthermore, liver and brain tissues were removed for histological analysis and biochemical evaluation.

### Biochemical assays

Plasma ammonia (NH4), serum sodium (Na+), and serum potassium (K+) levels, besides serum alkaline phosphatase (ALP) and gamma-glutamyl transferase (GGT) activities, were determined colorimetric kits using a spectrum commercial diagnostic kit supplied by biosystems, Barcelona, Spain. Liver tumor necrosis factor-alpha (TNF-α), interleukin-6 (IL-6), interleukin-12 (IL-12), interleukin-18 (IL-18) and cyclooxygenase (COX2) levels were measured by ELISA kits supplied by Creative Biolabs, USA (ITS-0622-CR184); Abcam, USA (ab234570); Elabscience, USA (E–EL-R0064); My BioSource, USA (MBS453975); and My BioSource, USA (MBS266603) respectively. Liver superoxide dismutase activity (SOD), and reduced glutathione (GSH) content in addition to malondialdehyde (MDA) level were determined using colorimetric kits purchased from bio diagnostic, Egypt (cat no. SD 2520, GR 2510, and MD 2528 respectively). Moreover, liver Paraoxonase 1 (PON1) activity was estimated using an ELISA kit obtained from My BioSource, USA (MBS453155).

### Assessment of cytochromes P450 gene expression and hepatic nuclear factor kappa-B expression

Total RNA was isolated from liver tissue homogenates using the RNeasy Micro Kit, which was purchased from Qiagen, Germany (cat. no. 74004), by the manufacturer’s instructions. The collected RNA was converted to complementary DNA using an RT-PCR kit (cat. no. 205311) that was purchased from Qiagen, Germany. Nuclear factor kappa-B (NF-κB) and cytochromes P450 (CYP450) were subjected to a quantitative real-time polymerase chain reaction (QPCR) using SYBR Green PCR master mix, which was obtained from Qiagen, Germany (cat. no. 204056) by the manufacturer’s instructions. GAPDH was used as a housekeeping gene. The primer sequences for each gene are displayed in Table 1. Quant Studio TM design and analysis software version 1.5.2 was utilized to perform quantification and analysis on a Quant Studio 3 real-time PCR machine that was purchased from Thermo Fisher Scientific, USA. The 2-ΔΔCt technique was utilised to compute the relative expression levels of the target genes.

**Table 1:** Primers sequences for RT-PCR analysis

Target gene	Primer sequences
NF-κB	F: 5ʹ-GGTTACGGGAGATGTGAAGATG-3ʹ
	R: 5ʹ-GTGGATGATGGCTAAGTGTAGG-3ʹ
CYP450	F: 5ʹ-TGCAGGTGGACTTTGAGAAC-3ʹ
	R: 5ʹ-TACAGGCTTCCAGGCTATCT-3ʹ
GAPDH	F: 5ʹ-ACT CCC ATT CTT CCA CCT TTG-3ʹ
	R: 5ʹ-CCC TGT TGC TGT AGC CAT ATT-3ʹ

The levels of brain norepinephrine (NE), epinephrine (EPI), dopamine (DA), serotonin (ST), and acetylcholine esterase (ACh) levels were determined using suitable ELISA kits obtained from My BioSource, USA (MBS704163); (MBS269993); (MBS725908), (MBS166089) and (MBS728879) respectively. Besides brain nitric oxide level was determined using a colorimetric diagnostic kit obtained from Bio diagnostic, Egypt (NO2532).

### Histological examination

Samples of the liver and brain taken from different groups were preserved in 10% buffered neutral formalin for a full day. After that, standard processing was applied to the fixed specimens to produce paraffin blocks. Subsequent blocks underwent serial sectioning into sections 4–5 µm thick for histological and immunohistochemical analysis. Haematoxylin and eosin (H&E) staining was applied to sections from each group.

### Statistical analysis

The SPSS (SPSS 25) was used in data analyses. Data were analyzed with a one-way analysis of variance (ANOVA) followed by a post hoc test (Duncan multiple range) for multiple comparisons. The data were expressed as mean ± standard error (SE). *P* values < 0.05 were considered statistically significant.

## Results

### Effect of BA and /or LDR on the alterations of ammonia, sodium and potassium levels and the activity of the hepatic enzymes

As shown in Table 2, it was found that the injection of TAA significantly enhances the increase in plasma level of ammonia and serum Na+ of rats accompanied by suppression in serum level of K+ compared to the control group (*P* < 0.05). However, the administration of BA and/or exposure to LDR promoted a significant decrease in the levels of Na+ and ammonia concomitant with significant elevation in the K+ concentration with respect to the TAA intoxicated groups in groups of animals BA, BA+LDR and LDR. The results also revealed that the injection of TAA into groups of animals (TAA, TAA+BA, TAA+LDR and TAA+LDR+BA), significantly enhanced the activity of liver enzymes (ALP and GGT) in the serum of rats compared to the control group. However, the administration of BA either alone or combined with LDR significantly suppressed ALP and GGT activities in groups (BA), (BA+LDR) and LDR) compared to the TAA group (*P* < 0.05) (Table 2).

**Table 2:** Changes of ammonia concentrations in plasma and Na+ and K+ levels and the activities of serum ALP and GGT in different groups of rats

Parameters group	Ammonia (μg/dl)	Na^+^ (mmol/L)	K^+^ (mmol/L)	ALP (U/L)	GGT (U/L)
Control	411 ± 0.99^a^	147 ± 2.1^a^	4.3 ± 0.14^a^	115 ± 1.1^a^	14.5 ± 0.72^a^
BA	407 ± 1.6^a^	144 ± 1.9^a^	5.1 ± 0.11^b^	112 ± 1.1^a^	12.1 ± 0.63^a^
LDR	405 ± 2.9^a^	144 ± 2.1^a^	4.9 ± 0.11^b^	116 ± 1.4^a^	14.1 ± 0.67^a^
BA+LDR	407 ± 2.1^a^	145 ± 1.6^a^	4.7 ± 0.07^b^	113 ± 1.3^a^	12.6 ± 0.83^a^
TAA	664 ± 3.5^f^	159 ± 1.2^b^	3.4 ± 0.07^d^	244 ± 7.1^e^	45 ± 0.52^d^
TAA+BA	447 ± 1.6^c^	145 ± 1.9^a^	4.2 ± 0.06^a^	148.7 ± 3.4^c^	25.5 ± 0.89^c^
TAA+LDR	460 ± 1.2^d^	144 ± 1.1^a^	3.9 ± 0.05^c^	177 ± 3.3^d^	26.5 ± 1.5^c^
TAA+BA+LDR	421 ± 0.9^b^	144 ± 2.1^a^	4.3 ± 0.07^a^	138 ± 2.4^b^	18.3 ± 0.59^b^

Values are represented as mean ± SE. Similar symbols within the same parameter mean non-significant while different symbols mean significant at *P* < 0.05 (Duncan’s test). Na+ and K+ levels in serum = mmol/l and Plasma ammonia concentration = ug/dl

*BA* Boswellic acids, *LDR* Low dose gamma radiation, *TAA* Thioacetamide

### Effect of BA and /or LDR on oxidative stress biomarkers

**Fig. 1:**
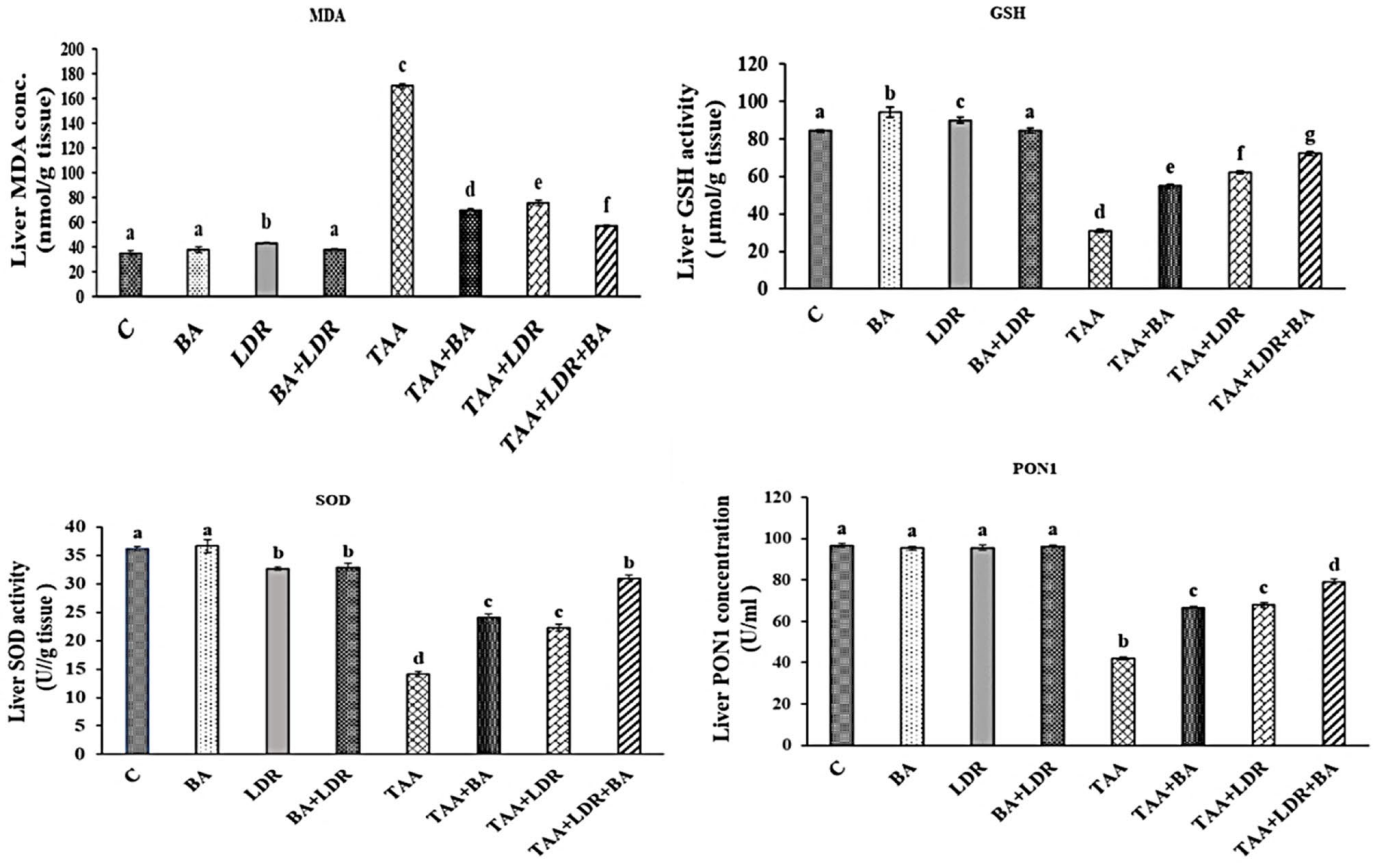
Changes in hepatic content of MDA and GSH and activities of SOD and PON1 in different groups of animals. Values are represented as mean ± SE. Similar symbols within the same parameter mean non-significant while different symbols mean significant at *P* < 0.05 (Duncan’s test). *BA* Boswellic acids, *LDR* Low dose gamma radiation, *TAA* Thioacetamide

As shown in Fig. 1, it was noted that the exposure to TAA was associated with a rise in hepatic MDA concentration accompanied by a decrease in the levels of hepatic GSH and activities of SOD and PON1 compared with the control group (*P* < 0.05). Meanwhile, administration of BA and /or exposure to LDR showed a significant decrease in the hepatic level of MDA accompanied by enhancement in the level of GSH and activities of SOD and PON1 in groups of animals TAA+BA, TAA+LDR compared to TAA group (*P* < 0.05).

### Effect of BA and /or LDR on the activity of the proinflammatory cytokines

**Fig. 2:**
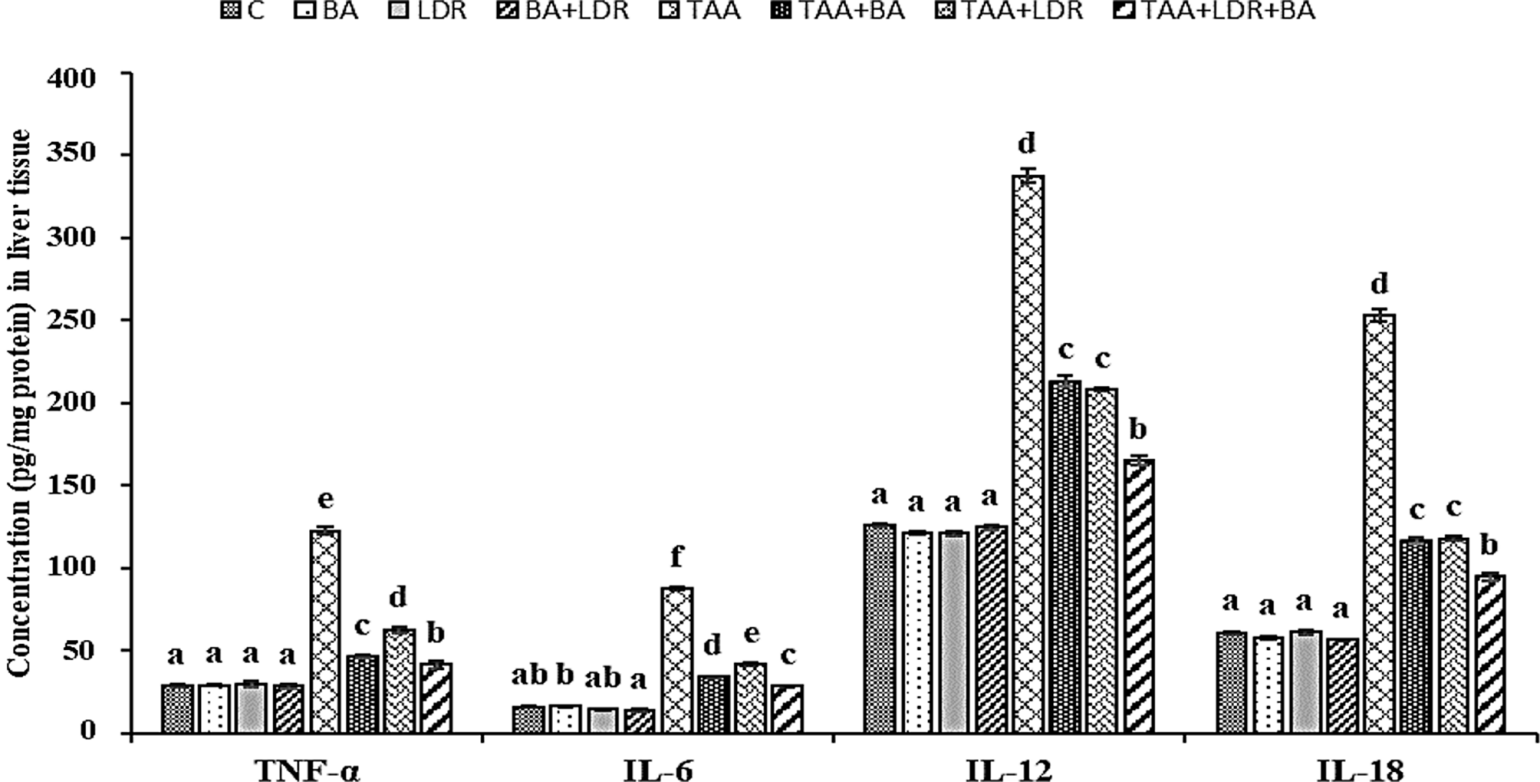
Effect of TAA, BA and BA+LDR on inflammatory biomarkers in hepatic tissue homogenate of rats in the different groups. Values are represented as mean ± SE. Similar symbols within the same parameter mean non-significant while different symbols mean significant at *P* < 0.05 (Duncan’s test). *BA* Boswellic acids, *LDR* Low dose gamma radiation, *TAA* Thioacetamide

Results in Fig. 2 indicated that the levels of hepatic TNF-α, IL-6, IL-12 and IL-18 of animals injected with TAA were significantly increased compared to the control group (*P* < 0.05). Meanwhile, Oral administration of BA alone and/or LDR exposure markedly suppressed the levels of proinflammatory cytokines (TNF-α & IL-6 & IL-12 and IL-18) in TAA injected groups (TAA+BA), (TAA+LDR), (TAA+BA+LDR) compared to TAA group (*P* < 0.05).

### Effect of BA and /or LDR on gene expressions of NF–kB and CYP450 and the level of Cox2

**Fig. 3:**
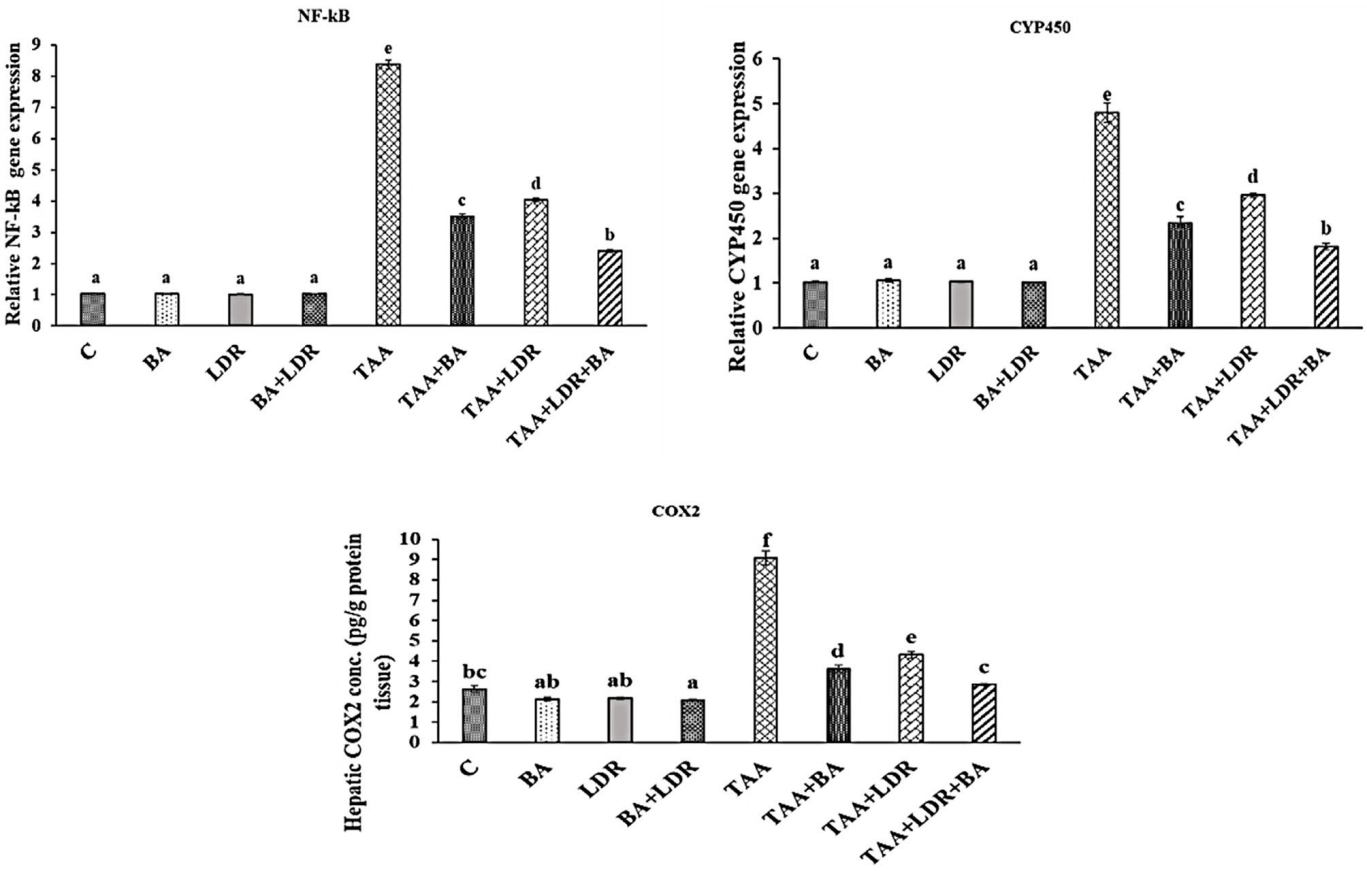
Effect of BA and/or LDR on the gene expression of NF–kB and CYP450 and level of Cox2 in the liver tissue of different animal’s groups. Values are represented as mean ± SE. Similar symbols within the same parameter mean non-significant while different symbols mean significant at *P* < 0.05 (Duncan’s test). *BA* Boswellic acids, *LDR* Low dose gamma radiation, *TAA* Thioacetamide

Regarding the gene expression of NF–kB and CYP450 and Cox2 levels in liver tissue homogenates in Fig. 3, it was found that TAA intoxication upregulated the mRNA expression of NF–kB and CYP450 genes and Cox2 level in liver tissue homogenate compared to the normal control group (*P* < 0.05). However, oral administration of BA and /or LDR significantly modulated the expression of NF–kB and CYP450 genes and levels of Cox2 in animal groups exposed to TAA (TAA+BA), (TAA+LDR), (TAA+BA+LDR) compared to TAA group (*P* < 0.05).

### Effect of BA and /or LDR on brain neurotransmitters biomarkers

**Fig. 4:**
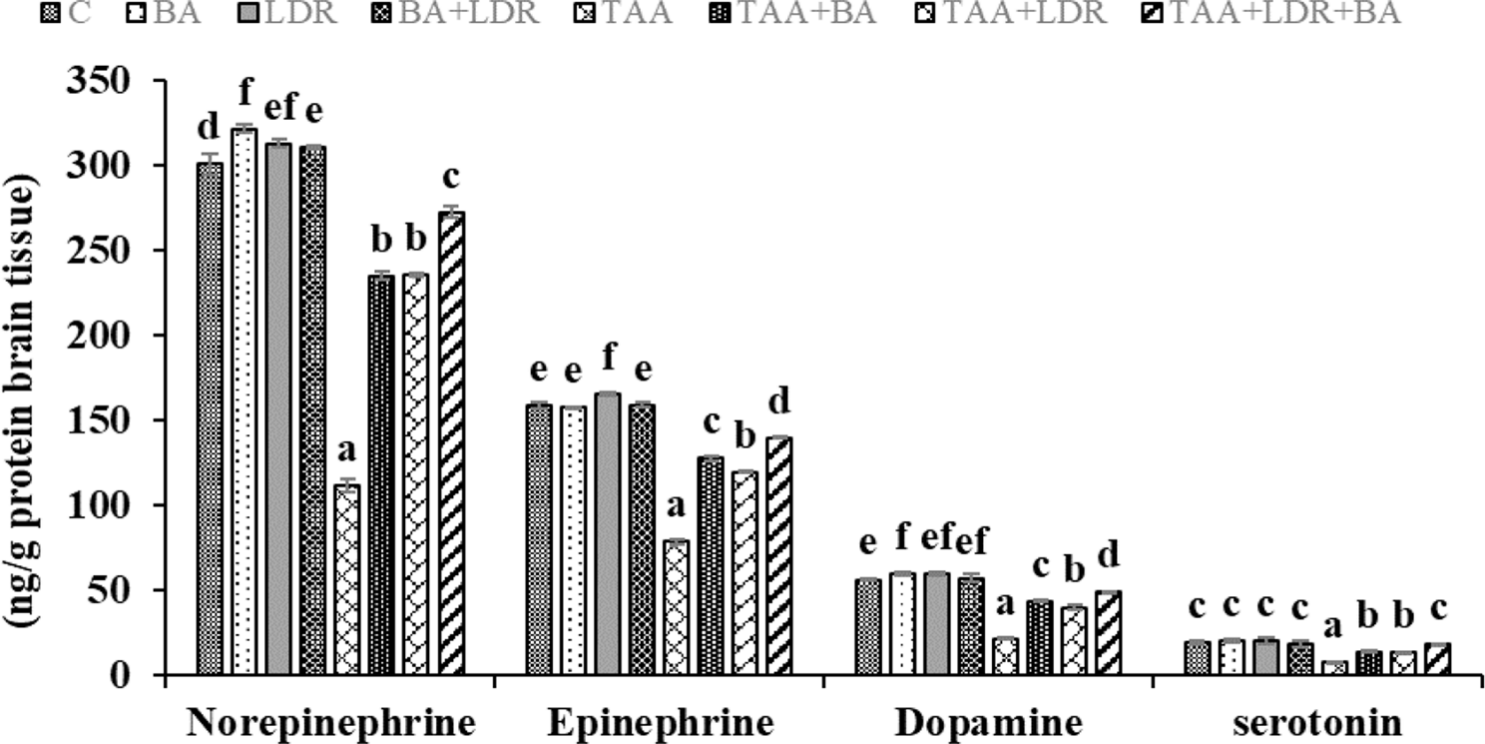
Changes in brain neurotransmitter level of animal rats in different groups. Values are represented as mean ± SE Similar symbols within the same parameter mean non-significant while different symbols mean significant at *P* < 0.05 (Duncan’s test). *BA* Boswellic acids, *LDR* Low dose gamma radiation, *TAA* Thioacetamide

From Fig. 4, it was found that injection of TAA showed a significant decrease in brain neurotransmitters; norepinephrine, epinephrine, dopamine and serotonin in respect to normal control (*P* < 0.05). However, the administration of BA and/or exposure to LDR significantly altered the levels of neurotransmitters in groups of animals (TAA+BA), (TAA+LDR) and (TAA+LDR+BA) compared to the TAA group (*P* < 0.05).

As shown in Table 3, it was observed that injection of TAA markedly provoked the production of brain ACH and NO compared to the control group (*P* < 0.05). On the other hand, oral administration of BA and/or LDR exposure significantly reduced the levels of ACH and NO in groups of animals injected with TAA (TAA+BA), (TAA+LDR), (TAA+LDR+BA) compared to TAA group (*P* < 0.05).

**Table 3:** Changes in brain NO level and neuro ACH activity of animal rats in different groups

Parameters group	ACH (U/mg tissue)	NO (nmol/mg tissue)
C	24.7 ± 1.2^a^	3.2 ± 0.05^a^
BA	21.8 ± 1.1^ab^	2.9 ± 0.06^a^
LDR	22.9 ± 0.5^ab^	3.1 ± 0.17^a^
BA+LDR	20.2 ± 0.7^b^	3.1 ± 0.03^a^
TAA	67.4 ± 1.5^f^	11.6 ± 0.45^b^
TAA+BA	43.3 ±.0.6^c^	4.9 ± 0.19^c^
TAA+LDR	46.5 ± 1.1^d^	5.3 ± 0.11^c^
TAA+LDR+BA	33.3 ± 0.54^e^	3.9 ± 0.19^d^

Values are represented as mean ± SE Similar symbols within the same parameter mean non-significant while different symbols mean significant at *P* < 0.05 (Duncan’s test)

*BA* Boswellic acids, *LDR* Low dose gamma radiation, *TAA* Thioacetamide

### Histological examination of liver

**Fig. 5:**
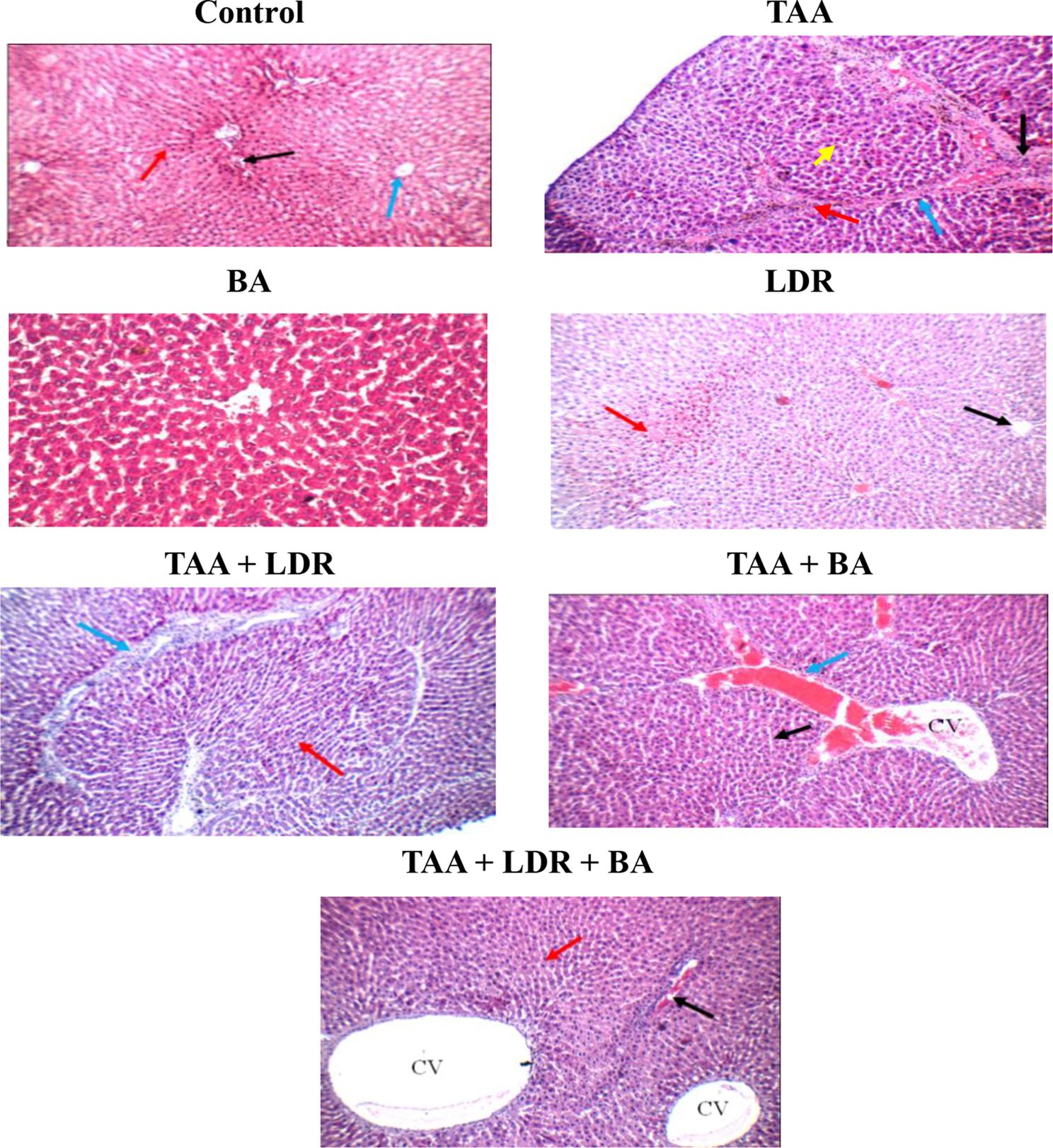
Photomicrograph of H&E-stained liver sections in all rat groups. Control: Photomicrograph of the liver section showing average portal tracts (black arrow), average central vein (blue arrow) and normal hepatocytes with well-defined nuclei and cell boundaries (red arrow). **TAA** Photomicrograph of the liver section showing expanded portal tract with mild inflammatory infiltration (black arrow) and thick fibrous septa with complete nodule formation (blue arrow). Presence of markedly pyknotic hepatocytes (yellow arrow) and distinct hemosiderin deposits (red arrow). **BA:** There was no histopathological alteration, and the normal histological structure of the central vein and surrounding hepatocytes was recorded. LDR: showing average central vein (black arrow), and normal hepatocytes except for the small number of necrotic hepatocytes (red arrow). **TAA+LDR:** showing thick fibrous septa with mild inflammatory infiltration (blue arrow), incomplete nodule formation (red arrow) and presence of normal hepatocytes. **TAA+BA:** showing mildly dilated congested central veins (CV) (blue arrow) and normal hepatocytes (black arrow). **TAA+LDR+BA:** showing average portal tract (black arrow)), markedly dilated central veins (CV) and normally shaped hepatocytes (red arrow) (H&E X 200)

As shown in Fig. 5, **liver of control group** showed average portal tracts with average portal veins and average hepatocytes in peri-portal area, and average central veins with average hepatocytes arranged in single-cell cords with average intervening blood sinusoids. **Liver of TAA group** showed expanded portal tracts with mild portal inflammatory infiltrate, scattered ciderophages, mildly dilated congested portal veins and markedly apoptotic hepatocytes in peri-portal area, thick fibrous septa with complete nodule formation, and mildly dilated congested central veins with areas of hemorrhage and scattered apoptotic hepatocytes in peri-venular area. **Liver of LDR group** showed mildly edematous portal tracts, mildly dilated congested portal veins and scattered apoptotic hepatocytes in peri-portal area, and mildly dilated congested central veins with markedly apoptotic hepatocytes in peri-venular area, small areas of necrosis, and eosinophilic plaque-like areas. **Liver of TAA+LDR group** showed expanded portal tracts with mild portal inflammatory infiltrate, mildly dilated congested portal veins and markedly apoptotic hepatocytes in peri-portal area, thick fibrous septa with complete nodule formation, and average central veins. **Liver of TAA+BA** showed expanded portal tracts with mild portal inflammatory infiltrate with thick septa, markedly dilated congested portal veins and scattered apoptotic hepatocytes in peri-portal area, and mildly dilated congested central veins and scattered apoptotic hepatocytes in peri-venular area. **Liver of TAA+LDR+BA:** liver showed mildly edematous portal tracts, mildly congested portal veins, short fibrous septa and average hepatocytes in peri-portal area, and markedly dilated central veins and scattered apoptotic hepatocytes in peri-venular area.

### Histopathological examination of brain

**Fig. 6:**
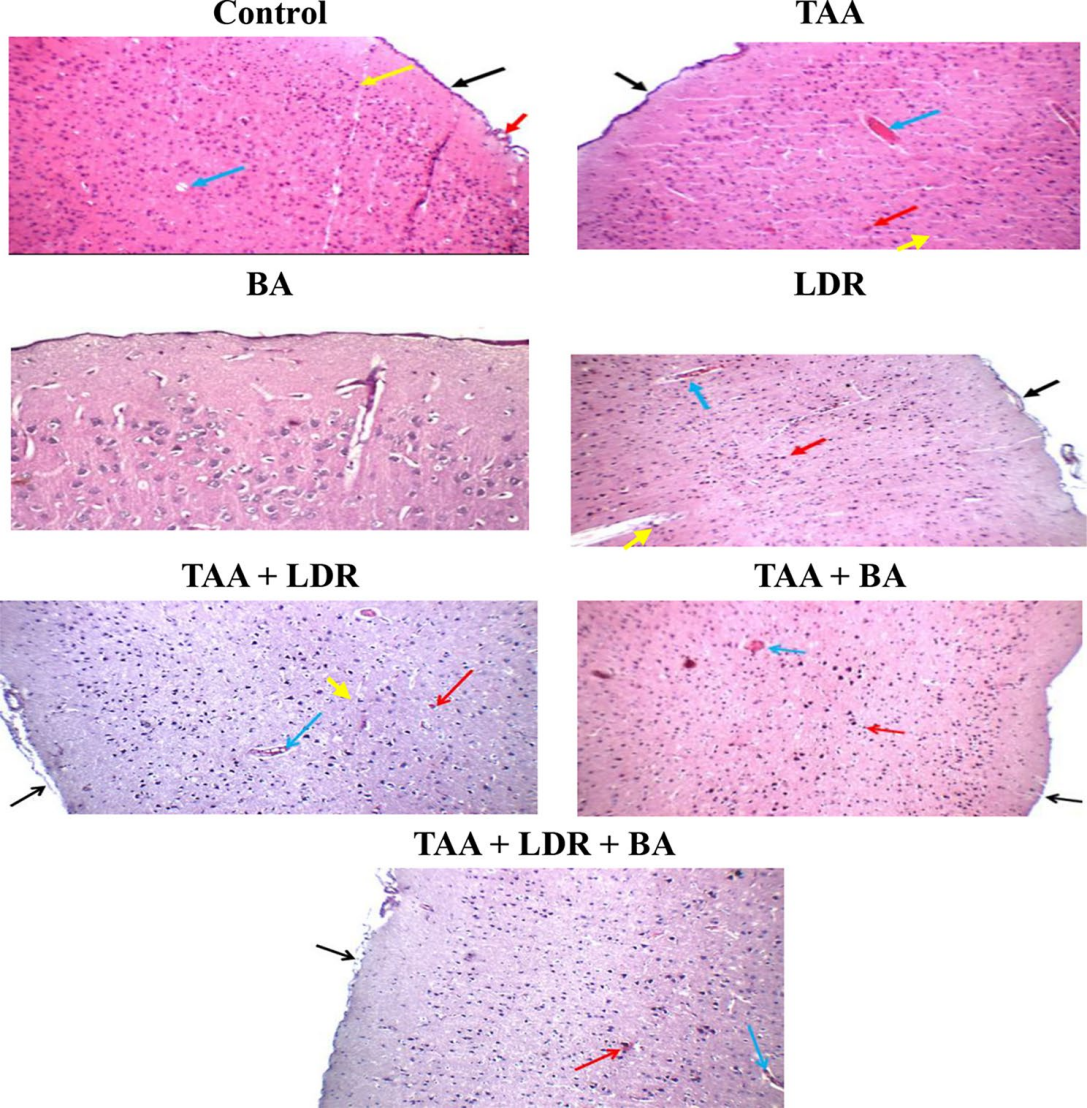
Photomicrograph of H&E-stained brain sections in all rat groups. Control: Photomicrograph of brain section showing regular meninges (black arrow), typical meningeal (red arrow) and intra-cerebral blood vessels (blue arrow), and normal cerebral cortex with normal sized and shaped neurons (yellow arrow). **TAA:** brain section showing average meninges (black arrow), mildly congested intra-cerebral blood vessels (blue arrow), cerebral cortex showing scattered degenerated neurons (yellow arrow), and eosinophilic plaque-like areas (red arrow**). BA:** There was no histopathological alteration and the normal histological structure of the meninges and cerebral cortex were recorded. **LDR:** showing average meninges (black arrow), mildly congested intra-cerebral blood vessels (blue arrow), and cerebral cortex showing normal neurons (red arrow) and scattered degenerated neurons (yellow arrow). **TAA+LDR:** showing average meninges (black arrow), mildly congested intra-cerebral blood vessels (blue arrow), and cerebral cortex showing normal neurons (yellow arrow) and scattered degenerated neurons (red arrow). **TAA+BA:** showing average meninges (black arrow), mildly congested intra-cerebral blood vessels (blue arrow), and cerebral cortex showing average neurons and glial cells (red arrow). **TAA+LDR+BA:** showing average meninges (black arrow), average intra-cerebral blood vessels (blue arrow), and cerebral cortex showing normal neurons and glial cells (yellow arrow) and scattered degenerated neurons (red arrow) (H&E X 200)

As shown in Fig. 6, **brain of control group** showed average meninges, average meningeal and intra-cerebral blood vessels, average cerebral cortex with average neurons and average astrocytes, and striatum showed average neurons and average glial cells in fibrillary background. Brain of **TAA** showed average meninges with mildly congested sub-meningeal and intra-cerebral blood vessels, cerebral cortex with scattered degenerated neurons, average glial cells and eosinophilic plaque-like areas, and striatum showed scattered degenerated neurons, average glial cells and eosinophilic plaque-like areas. **Brain of LDR group** showed average meninges, mildly congested intra-cerebral blood vessels, cerebral cortex with scattered degenerated neurons, average glial cells and eosinophilic plaque-like areas, and striatum showed scattered degenerated neurons, average glial cells and eosinophilic plaque-like areas. Brain of **TAA+LDR group** showed average meninges with small sub-meningeal hemorrhage, mildly congested intra-cerebral blood vessels, cerebral cortex with markedly degenerated neurons and markedly degenerated glial cells, and striatum showed average neurons, average glial cells and markedly congested blood vessels. **Brain of TAA+BA** showed average meninges, cerebral cortex showed scattered degenerated neurons, average glial cells, eosinophilic plaque-like areas and mildly congested intra-cerebral blood vessels, and striatum showed average neurons, average glial cells and markedly congested blood vessels. Brain of **TAA+LDR+BA:** brain showed average meninges, cerebral cortex showed scattered degenerated neurons, average glial cells, eosinophilic plaque-like areas and mildly congested intra-cerebral blood vessels, and striatum showed average neurons, average glial cells and mildly congested blood vessels.

### Histopathological Examination of Hippocampus

**Fig. 7:**
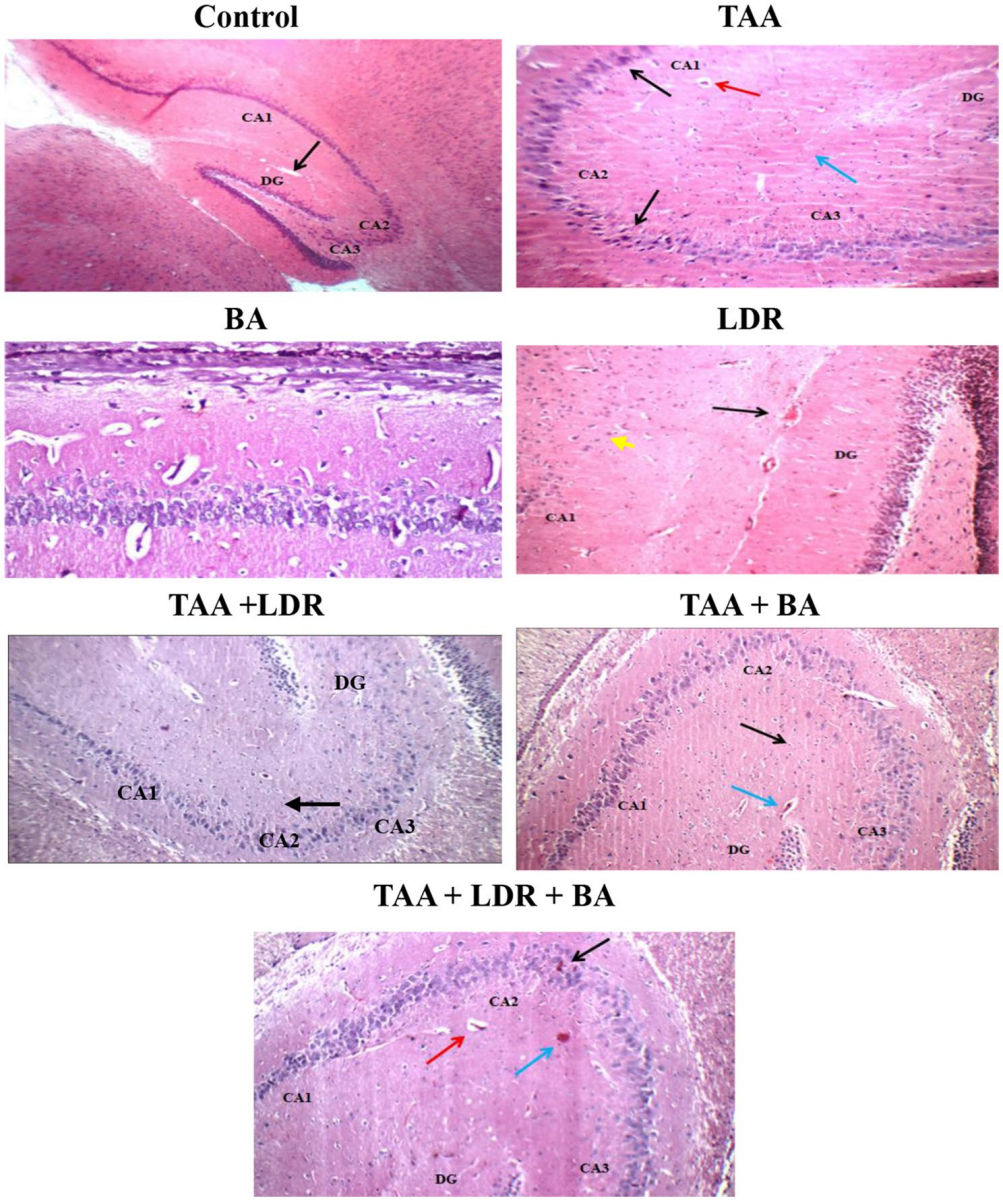
Photomicrograph of H&E-stained brain sections in all rat groups. Control: hippocampus showing average Cornu Amonis (CA1), (CA2), (CA3), average dentate gyrus (DG), and average blood vessels (black arrow) in (DG). **TAA:** showing scattered degenerated neurons (black arrow) in Cornu Amonis (CA1), (CA2), average inter-neuron area (blue arrow), and average blood vessels (red arrow). **LDR:** showing average dentate gyrus (DG) with mildly congested blood vessels (black arrow) and normally shaped and sized neurons. **BA:** There was no histopathological alteration and the normal histological structure of the hippocampus and striatum of the cerebrum. **TAA+LDR:** showing average Cornu Amonis (CA1), (CA2), (CA3), normal dentate gyrus (DG), and regular inter-neuron area (black arrow) in (DG). **TAA+BA:** showing average Cornu Amonis (CA1), (CA2), (CA3), normal dentate gyrus (DG), regular inter-neuron area (black arrow), and normal blood vessels (blue arrow) in (DG). **TAA+LDR+BA:** showing normal Cornu Amonis (CA1), (CA2), scattered degenerated pyramidal neurons (black arrow) in (CA3), eosinophilic plaque-like areas (blue arrow), and regular blood vessel (red arrow) (H&E X 200)

As shown in Fig. 7, **hippocampus of control group** showed average Cornu Amonis (CA1), (CA2), (CA3), average dentate gyrus (DG), average pyramidal neurons, average inter-neuron area, and average blood vessels. **Hippocampus of TAA group** showed scattered degenerate pyramidal neurons in (CA1), (CA2), (CA3), eosinophilic plaque-like areas in (CA2), and average dentate gyrus (DG) with mildly congested blood vessels. **Hippocampus of LDR** showed markedly degenerate pyramidal neurons and eosinophilic plaque-like areas in (CA3), and average dentate gyrus (DG) with mildly congested blood vessels. **Hippocampus of TAA+LDR** group showed average pyramidal neurons in (CA1), (CA2), (CA3), and in (DG), average inter-neuron area, and mildly congested blood vessels in (CA1), and in (DG)**. Hippocampus of TAA+BA group** showed average pyramidal neurons in (CA1), (CA2), (CA3), and in (DG), eosinophilic plaque-like areas in (CA1), and average blood vessels**. Hippocampus of TAA+LDR+BA** showed scattered degenerated pyramidal neurons in (CA2), eosinophilic plaque-like areas in (CA1), (CA2), (CA3), and in (DG), and mildly congested blood vessels in (DG).

## Discussion

Individuals suffering from severe liver complications can experience hepatic encephalopathy (HE), a significant neurological condition. In this context, acute liver failure has been experimentally induced using TAA to mimic the pathophysiological characteristics of acute liver disorders observed in humans [15]. The findings of the current study indicated that TAA led to liver dysfunction and brain discomfort. Following TAA treatment, there was a significant rise in serum liver enzyme activity in the animals, as well as an increase in ammonia levels. These results align with those reported by Jamshidzadeh et al. [16]. Similar research has demonstrated that TAA can cause hepatocellular damage to membranes, leading to hepatic fibrotic changes and a marked increase in liver enzyme activity. The elevated liver enzyme levels post-TAA treatment suggest reduced structural and functional integrity of liver cells, along with liver cellular leakage [17]. In this study, hyperammonemia might have contributed to the liver’s impaired detoxification processes and the alkalosis-related reduction in ammonia excretion through urine, which could have resulted in neurotoxicity due to direct ammonia exposure [18]. Additionally, the increase in plasma ammonia levels observed in this study is supported by findings from Dhorajiya and Galani [19], as well as Ahmed et al. [20].

Once in circulation, microsomal CYP2E1 activates and metabolizes TAA within hepatocytes, leading to the production of highly toxic metabolites [21]. As noted by Swapna et al. [22], these metabolites can cause hepatic necrosis, inflammation, and oxidative stress by binding to tissue macromolecules [23]. The resulting liver damage results in a decreased number of functional hepatocytes and impairs the liver’s ability to remove harmful substances from the bloodstream.

The study revealed that administering BA to rats or exposing them to fractionated low-dose radiation (LDR) notably reduced the activities of serum ALP and GGT, and significantly decreased ammonia levels. These results imply that BA offers protection against hepatotoxins, likely due to its antioxidant properties that help preserve the structural integrity of hepatocellular membranes [24]. In cases of hyperammonemia, BA supplementation effectively stabilized the membranes and significantly lowered ammonia levels. This effect may be linked to the enhancement of hepatic microcirculation by BA, which could contribute to maintaining liver structure and boosting its detoxification capabilities. These findings align with prior studies on the hepatoprotective effects of LDR [16].

Additionally, the potential link between LDR-induced hormetic responses in cells and its ability (up to 0.5 Gy) to increase cellular resistance to oxidative stress evidenced by improved liver enzyme levels could account for the beneficial effects of LDR. Compared to the control group, rats challenged with TAA exhibited a significant rise in the lipid peroxidation marker (MDA) and a substantial decrease in the activities of SOD, paraoxonase-1, and GSH levels. This indicates a disruption in the redox balance within liver and brain tissues. Research by Murthy et al. [25] and Túnez et al. [26] confirmed that TAA triggers oxidative stress by generating reactive oxygen species (ROS) and lipid peroxidation. The metabolism of TAA results in the production of harmful metabolites that can denature cellular macromolecules, particularly lipids, leading to lipid peroxidation and its metabolites.

Our findings indicate a notable decline in the activity of superoxide dismutase (SOD) and reduced glutathione (GSH) levels in both the liver and brain after TAA exposure. These results align with the work of Ahmed et al. [20] and Abdel-Rafei et al. [23], who found that treatment with boswellia serrata (BA) and/or low-dose radiation (LDR) decreased malondialdehyde (MDA) levels while enhancing antioxidant defenses (GSH, paraoxonase-1, and SOD) in the livers of their test subjects. The beneficial effects of BA suggest its potential as an antioxidant in neutralizing free radicals generated by TAA. Numerous studies have documented the antioxidant and radical-scavenging properties of BA [7]. There seems to be a relationship between the molecular structure of BA and its antioxidant activity. Additionally, the findings from Mansour et al. [27], Fahmy et al. [28] and El-Ghazaly et al. [29] who reported that rats subjected to whole-body LDR exhibited restored levels of endogenous GSH, SOD, and catalase (CAT) while significantly reducing MDA, supporting the antioxidant effect of LDR found in our research.

The increase in antioxidant enzyme activity is likely due to the induction of their synthesis following LDR exposure. It has been proposed that LDR enhances thioredoxin (TRX) activity at the cellular level, which may be responsible for restoring GSH levels in the liver and brain [30]. TRX, beyond its role as an endogenous antioxidant, is vital for regulating cellular redox status and facilitates the transport of cystine into cells that aid GSH production [23]. Excessive oxidative stress can activate nuclear factor kappa B (NF-κB), a transcription factor implicated in processes such as inflammation and apoptosis, leading to the release of various pro-inflammatory cytokines [31]. Consistent with the conclusions drawn by Ali et al. [32], our study found significantly elevated levels of inflammatory mediators including TNF-α, IL-6, IL-12, and IL-18 following TAA exposure. The liver is considered essential in modulating cytokine production and activity, influenced by early pro-inflammatory cytokines released by macrophages. As a hepatotoxin, TAA induces hepatic macrophages (Kupffer cells) to produce pro-inflammatory and inflammatory mediators like TNF-α, IL-6, cyclooxygenase-2 (Cox-2), and NF-κB, which play a critical role in hepatic inflammation [33]. In contrast, the administration of BA reduced the synthesis of chemokines and inflammatory cytokines. This suggests that BA may modulate the immune response, contributing to its protective effects in rats. Our findings demonstrate that both LDR and BA significantly decreased levels of TNF-α, IL-6, IL-12, and IL-18, along with a reduction in Cox-2 levels, which aligns with earlier studies [34]. Moreover, our results indicated a correlation between hyperammonemia and elevated nitric oxide (NO) and acetylcholinesterase (AChE) activity.

These results correspond with findings from Garcia-Ayllon et al. [35] The notable reduction in AChE levels, a neurotransmitter critical for cognitive function, coincided with increased AChE activity, as noted in studies involving rats with acute and subacute hyperammonemia. Mansour et al. [4] concluded that these changes are likely due to alterations in membrane structure that expose more catalytic sites, rather than modifications to the enzymes themselves. Boswellia serrata oleo gum has also been shown to reduce NO production, and compounds that lower NO levels in liver tissue are associated with hepatoprotective properties [36]. It appears that boswellia serrata may exert protective effects on liver function through its ability to reduce NO production [7]. Furthermore, the inhibitory effect of BA on acetylcholinesterase activity may be linked to the free hydroxyl group at C-3 and the keto group at C-11 of its ursane skeleton [37]. Hepatic encephalopathy is characterized by memory impairments [38] and disturbances in sensory and motor functions, which can be attributed to fluctuations in neurotransmitter levels [39].

Additionally, the pathophysiology of hepatic encephalopathy may involve an increase in the activity of metabolizing enzymes, which subsequently alters monoaminergic synaptic activity [8]. The findings from this study indicated that exposure to thioacetamide resulted in changes in the brain levels of neurotransmitters such as dopamine, norepinephrine, epinephrine, and serotonin when compared to control values. This aligns with research by Said et al. [40] and Ismail and El-Sonbaty [41], which showed that rats subjected to whole-body γ-irradiation experienced altered neurotransmitter levels associated with reduced brain electrical activity. These results further support Herrera et al. [42] suggestion that changes in monoamine synthesis may underlie these alterations. Specifically, the absorption of tryptophan was connected to the observed changes in serotonin, while decreased absorption of L-tyrosine may explain the reduction in the production of dopamine, norepinephrine, and epinephrine. The administration of BA either alone or alongside low-dose radiation (LDR), effectively restored the neurotransmitter disturbances induced by thioacetamide, suggesting a beneficial impact of BA on hepatic encephalopathy.

In regard of the relatively low pharmacological properties of BA, this study suggests that combining low-dose gamma radiation (LDR) with BA may enhance its pharmacological effectiveness. The findings indicate that this combination could serve as a promising strategy to alleviate hyperammonemia associated with acute or chronic liver damage. However, further research is necessary to improve the bioavailability of BA.

## Data Availability

No datasets were generated or analysed during the current study.
